# Intraoperative diagnosis of solitary cecal diverticulum not requiring surgery: is appendectomy indicated?

**DOI:** 10.1186/s13017-015-0057-y

**Published:** 2016-01-04

**Authors:** Renol M. Koshy, Abdelrahman Abusabeib, Saif Al-Mudares, Mohamed Khairat, Adriana Toro, Isidoro Di Carlo

**Affiliations:** Department of General Surgery, Hamad General Hospital, 3050 Doha, Qatar; Department of Surgery, Patti Hospital, Patti (ME), Italy; Department of Surgical Sciences and Advanced Technologies, “G.F. Ingrassia” University of Catania, Catania, Italy

**Keywords:** Solitary, Cecum, Diverticulum, Appendectomy

## Abstract

**Aim:**

To compare experience with solitary cecal diverticulum (SCD) with literature on the indication for appendectomy in cases of solitary cecal diverticulitis.

**Methods:**

We retrospectively reviewed all cases of SCD in our institution from September 2011 to March 2013. Data on sex, age, ethnic origin, presence of pain in the right iliac fossa, duration of symptoms, diagnosis, management, intraoperative findings, histologic examination, hospital stay, complications, and follow-up were reviewed and analyzed. We compared this to related literature reported between 2000 and 2015.

**Results:**

In the study period, 10 patients presented with an SCD. Male sex and Asian origin were predominant. All patients had pain in the right iliac fossa, with a duration of 2–5 days. In nine cases the diagnosis was made by clinical examination and laboratory testing. One patient who had undergone a previous appendectomy was diagnosed with SCD by computed tomography. This last patient was treated conservatively, four patients were treated with resection of the cecum “en bloc” with the last jejunal loop and appendix, and the other five patients were treated with appendectomies. Two patients had minor complications. All patients were followed up for a minimum of 12 to a maximum of 24 months. No recurrence was recorded in either the case treated conservatively or the cases treated by appendectomies.

**Conclusions:**

In cases of operative but conservative treatment for SCD, appendectomy could be justified to avoid misdiagnosis in case of future episodes of solitary cecal diverticulitis.

## Background

The cecal diverticula described for the first time by Potier in 1912 [1] remain a rare entity, especially if solitary, with an incidence between 1:50 and 1:300 of that of appendicitis [2]. The incidence of solitary cecal diverticulum (SCD) in North America is low at about 1–2 %; in contrast, SCD is more common in the Orient, accounting for 43–50 % of all cases of colonic diverticulosis [3].

Pain in the right iliac fossa (RIF) is a common presentation to the emergency department of most hospitals. Preoperative diagnosis is invariably difficult. The most common clinical misdiagnosis of diverticular disease of the right colon is acute appendicitis [4], and it is then on the operating table that we are faced with the reality of the actual diagnosis. More than 70 % of patients with cecal diverticulitis underwent a surgical procedure with an erroneous indication [5]. Other differential diagnoses to consider are urinary tract infection, ureteric colic, gastroenteritis, pelvic inflammatory disease, Crohn's disease [6], colonic malignancy, perforated foreign body reaction, and ileocecal tuberculosis [7]. The correct diagnosis is very important because acute diverticulitis of the right colon without complications can be treated medically [8].

When patients are subjected to a surgical procedure in the presence of an SCD that affected the patient but did not require surgical treatment, the necessity of then performing an appendectomy (AP) is still debatable. The aim of the present study is to retrospectively report our personal experience with SCD, and to compare this with a review of the literature focusing on the indication of AP in the presence of cecal diverticulitis not requiring surgery.

## Materials and methods

A retrospective analysis was performed on patients admitted to the Hamad General Hospital of Doha, Qatar from September 2011 to March 2013 with pain in the RIF. Sex, age, ethnic origin, duration of symptoms, diagnosis, management, intraoperative findings, histologic examination, length of hospital stay, complications, and follow-up of all patients affected by SCD were reviewed.

### Literature review

An extensive search for relevant literature between 2000 and May 07, 2015 was carried out using MEDLINE (PubMed) and Google Scholar with the language restricted to English, Italian, and French. The keywords used for the search were: ‘Right sided colon diverticulitis’, ‘caecum diverticulitis’, and ‘solitary cecum diverticula’. These keywords were used individually or with the Boolean operator ‘AND’.

We included articles that reported patient number, sex, age, duration of symptoms, diagnosis, type of surgical procedure, pathological report, compliance, recurrence, histopathologic examinations, length of hospital stay, and follow-up. Studies that did not clearly meet the inclusion criteria were excluded.

## Results

In the 18 months from September 2011 to March 2013, 2982 patients were evaluated and operated on for appendicitis at the Hamad General Hospital Department of Surgery. Ten of these patients were diagnosed with SCD with a ratio of 298.2:1, giving an incidence of SCD of 0.3 % or 1 in 300 APs. Nine of the 10 SCD patients were male, giving a 9:1 male to female ratio. All SCD patients were aged 19–40 years (mean age 30.4 years). Regarding nationality of the SCD patients, all patients were Oriental; there was one Indian, two Egyptians, one Sri Lankan, one Qatari, two Filipino, two Bangaladeshi, and one Syrian. The SCD patients presented for the first time to the Emergency Department with localized RIF pain of 2–5 days duration. All SCD patients except one were diagnosed with acute appendicitis on clinical examination. The diagnosis of appendicitis was based on the patients’ clinical presentation, and supported by a leukocytosis typical of acute appendicitis. However, one patient with pain for 5 days and a history of AP underwent a computed tomography (CT) scan and was managed conservatively; for this patient, a diagnosis of SCD was made preoperatively. Diagnosis of SCD for the remaining patients was made at the time of laparoscopy.

Of the 10 SCD patients, one had had a previous AP and was treated conservatively with antibiotic therapy, five patients were managed with AP alone, and four underwent an ileocecal resection. These last four patients were explored laparoscopically, and in all patients the surgery was converted to open surgery for the resection. The five patients managed with laparoscopic AP presented with an inflamed diverticulum that had not perforated; the appendix in these patients was removed to facilitate the diagnosis in case of secondary episode of RIF inflammation. All nine patients operated on (90 %) had a cecal mass; in four patients the flogosis did not permit us to distinguish the cecum from the appendix or the diverticulum, while in the other five patients it was still possible to identify the appendix and also the inflamed cecal diverticulum even in the presence of a cecal mass. In two of 10 cases (20 %) the cecal mass was located medially above the ileocecal junction, in four cases (40 %) the cecal mass was anterior, and in two cases (20 %) cases the cecal mass was located laterally. Two of 10 patients (20 %) had a perforation of their diverticulum; in one patient (10 %) this was treated with conservative antibiotic therapy, and in the other patient (10 %) this was treated with an ileocecectomy. All patients, including the nine that received surgery and the one that was treated conservatively, received intravenous antibiotics and gradually progressed on to a normal diet. The hospital stay varied from 4 to 10 days. Two patients had complications: one had urinary retention and another had a wound infection. All patients were given a follow-up examination, and all patients had no complaint after 1 month. All of these SCD patients have been followed up for a minimum of 12 to a maximum of 24 months, and no recurrence has been recorded in any case. However, the follow-up is limited as many of these patients are contractors who return to their native countries when they finish their period of work.

The histopathologic examinations of all four resected ileocecal specimens were reported as true solitary cecal diverticulitis. All nine appendices were reported as normal, including the four with the ileocecal specimen (Table 1).

**Table 1:** Main data of the patients affected by cecum diverticulum during the period of study

#	N. (N)	Duration of symptoms	Diagnosis	Management	Findings	Histopathology	Hosp days	Compl.
	Age/Sex							
1	AC. (I)	2 days	IO	Ileo-cecectomy	Cecal mass- anterior + perf.	SCD	10	Urinary retention
	39/M							
2	AH. (E)	2 days	IO	Ileo-cecectomy	Cecal mass- medial above ICJ	SCD	7	Wound infection
	24/M							
3	LN. (SL)	3 days	IO	Ileo-cecectomy	Cecal mass- medial above ICJ + perf.	SCD	4	None
	19/M							
4	AM. (E)	5 days + h/o Appendectomy	CT scan Abdomen	Conservative	CD + localized perforation	NA	5	None
	40/M							
5	MS. (Q)	2 days	IO	Ileo-cecectomy	Cecal mass- lateral	SCD	5	None
	22/M							
6	CM. (F)	2 days	IOL	Appendectomy	Cecal mass	N. App	6	None
	36/M							
7	AN. (B)	4 days	IOL	Appendectomy	Cecal mass- anterior	N. App	3	None
	29/M							
8	FT. (B)	1 day	IOL	Appendectomy	Cecal mass- anterior	N. App	2	None
	27/F							
9	MK. (S)	2 days	IOL	Appendectomy	Cecal mass- lateral	N. App	2	None
	31/M							
10	JH. (F)	3 days	IOL	Appendectomy	Cecal mass- anterior	N. App	3	None
	37/M							

*N* nationality, *I* Indian, *E* Egyptian, *SL* Sri Lankan, *Q* Qatari, *F* Filipino, *B* Bangaladeshi, *S* Syrian, *IO* intraoperative, *IOL* intraoperative laparoscopy, ICJ: ileocecal junction, *SCD* solitary cecum diverticulum, *NA* not available, *N. App* normal appendix

### Literature review

**Fig. 1:**
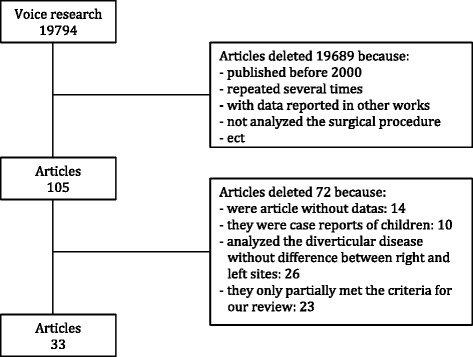
Algorithm used to screen the literature

A total of 19,794 published studies were screened from the sources listed. After examination of all titles, 19,689 papers were excluded as not relevant for reasons including being published before 2000, information repeated several times, containing data reported in other works, and the surgical procedure not being reported. Among the remaining 105 studies, the following were excluded: 14 articles were without data, 10 were case reports on children, 26 analyzed the diverticular disease without differentiating between the right and left sites, and 23 only partially met the inclusion criteria for our review (Fig. 1). A final total of 33 studies were included in the present study [2, 6, 7, 9–37].

In these 33 studies, a total of 1137 patients were analyzed. There were 643 males (56.5 %) and 477 females (41.9 %), excluding 18 patients (1.6 %) whose sex was not reported ([24], [37]). The mean patient age was 43.7 years. Only 22 studies reported the duration of symptoms, giving a mean of 2.9 days. The diagnosis was performed intraoperatively, or via CT scan or ultrasound (Table 2).

**Table 2:** Characteristics of patients with solitary cecum diverticulum and methods of diagnosis in the literature

Years	Authors	Pt	Male	Female	Mean Ages	Mean of duration of symptoms (days)	Diagnosis
2001	Law [9]	37	29	8	42	NR	NR
2003	Fang [10]	97	59	38	48	NR	CT scan + barium enema + intraoperative
2004	Papaziogas [11]	8	6	2	54	1,5	US + Intraoperative
2006	Connolly [6]	3	1	2	35	1,3	Intraoperative
2006	Yang [12]	113	74	39	44	NR	Intraoperative + CT scan
2006	Basili [13]	3			37	NR	US
2006	Ruiz-Tovar [14]	5	0	5	50	5,5	Intraoperative
2007	Griffiths [7]	1	1	0	67	1,0	Intraoperative
2007	Kurer [15]	1	1	0	26	3,0	Intraoperative
2007	Hildebrand [16]	16	12	4	60	3,0	Intraoperative
2007	Leung [17]	74	35	39	35	NR	Intraoperative
2007	Lee [18]	90	25	65	37	NR	US + CT scan + intraoperative
2008	Karatepe [19]	4	1	3	27	2,5	Intraoperative
2009	Kachroo [20]	1	0	1	63	3,0	CT Scan + US
2009	Malek [2]	1	0	1	34	4,0	Intraoperative
2009	Telem [21]	1	0	1	52	NR	TC scan
2009	Cole [22]	1	1	0	61	NR	Intraoperative
2009	Kumar [23]	2	1	1	32	11,0	Intraoperative
2009	Butt [24]	1	1	0	20	2,0	TC scan
2010	Matsushima [25]	110	61	49	43	NR	US + CT scan + intraoperative
2010	Kim [26]	158	99	59	40	2,7	NR
2011	Paramythiotis [27]	1	0	1	42	0,5	Intraoperative
2011	Radhi [28]	15	6	9		NR	TC scan + intraoperative
2012	Uwechue [29]	1	0	1	71	NR	Intraoperative
2012	Kwon [30]	59	42	17	44	NR	CT Scan + US
2012	Issa [31]	15			52	4,0	TC scan
2011	Tan [32]	68	46	22	43	NR	Intraoperative + CT scan
2013	Tan [33]	226	123	103	49	NR	CT scan + Intraoperative
2013	Kroening [34]	1	1	0	35	0,3	Intraoperative
2013	Gilmone [35]	1	0	1	26	1,0	CT scan
2013	Kahveci [36]	1	0	1	55	1,0	US
2015	Cristaudo [37]	13	8	5	44	4,3	1 US, 9 TC scan ,3 IO
2015	Our article	10	10	0	30	2,6	CT scan + Intraoperative
		1138	643	477	43,7	2,9	

*Pt* number of patients, *NR* not reported, *CT* computed tomography, *US*: ultrasound

The treatment for SCD was antibiotic therapy in 523 patients (46.49 %), AP in 202 patients (17.95 %), diverticulectomy (DV) in 58 patients (5.15 %), AP + DV in 38 patients (3.38 %), AP + excision of diverticulum in one patient (0.09 %), suture of diverticulum in two patients (0.18 %), suture of cecum in one patient (0.09 %), cecum resection in two patients (0.18 %), AP + amputation of the cecal pole in one patient (0.09 %). Ascending (limited right) hemicolectomy was reported in one patient (0.09 %). Right hemicolectomy and ileo-transverse anastomosis was performed in 222 patients (19.73 %); ileocecal resection and anastomosis was performed in 55 patients (1.51 %). Yang et al. reported on 17 patients (1.51 %) who were not divided into right hemicolectomy and ileo-transverse anastomosis and ileocecal resection and anastomosis [33]. Abscess drainage was performed in two patients (0.18 %). Kim et al. did not report the treatment in 13 patients (1.15 %) (Table 3) [26].

**Table 3:** Treatment of solitary cecum diverticulum

Years	Authors	Pt	AT	AP	DV	AP + DV	AP + EDV	SDV	SC	CR	AP + ACP	RH + ITA	ICR	LRH	AD
2001	Law [9]	37	--	--	1	--	--	1	--	--	--	35	--	--	--
2003	Fang [10]	97	18	36	9	--	--	--	--	--	--	34	--	--	--
2004	Papaziogas [11]	8	--	--	4	1	--	1	--	--	--	--	2	--	--
2006	Connolly [6]	3	--	--	--	2	--	--	1	--	--	--	--	--	--
2006	Yang [12]	113	56	32	--	8	--	--	--	--	--	17	--	--	
2006	Basili [13]	3	--	--	1	--	--	--	--	--	--	2	--	--	--
2006	Ruiz-Tovar [14]	5	1	--	--	--	--	--	--	--	--	1	3	--	--
2007	Griffiths [7]	1	--	--	--	--	--	--	--	--	--	1	--	--	--
2007	Kurer [15]	1	--	--	--	--	--	--	--	--	--	1	--	--	--
2007	Hildebrand [16]	16	--	--	--	--	--	--	--	--	--	10	6	--	--
2007	Leung [17]	74	--	36		8	--	--	--	--	--	14	16	--	--
2007	Lee [18]	90	28	16	40	--	--	--	--	--	--	6	--	--	--
2008	Karatepe [19]	4	--	--	1	2	1	--	--	--	--	--	--	--	--
2009	Kachroo [20]	1	--	--	--	--	--	--	--	--	--	1	--	--	--
2009	Malek [2]	1	--	--	--	--	--	--	--	--	--	--	1	--	--
2009	Telem [21]	1	1	--	--	--	--	--	--	--	--	--	--	--	--
2009	Cole [22]	1	--	--	--	--	--	--	--	--	--	1	--	--	--
2009	Kumar [23]	2	--	--	--	--	--	--	--	1	--	1	--	--	--
2009	Butt [24]	1	1	--	--	--	--	--	--	--	--	--	--	--	--
2010	Matsushima [25]	110	100	7	2	--	--	--	--	--	--	1	--	--	--
2010	Kim [26]	158	134	--	--	10	--	--	--	--	--	--	--	--	1
2011	Paramythiotis [27]	1	--	--	--	--	--	--	--	--	--	--	--	1	--
2011	Radhi [28]	15	--	--	--	--	--	--	--	--	--	15	--	--	--
2012	Uwechue [29]	1	--	--	--	--	--	--	--	1	--	--	--	--	--
2012	Kwon [30]	59	--	--	--	--	--	--	--	--	--	32	27	--	--
2012	Issa [31]	15	15	--	--	--	--	--	--	--	--	--	--	--	--
2011	Tan [32]	68	--	35	--	4	--	--	--	--	--	29	--	--	--
2013	Tan [33]	226	153	38	--	3	--	--	--	--	--	32	--	--	--
2013	Kroening [34]	1	--	--	--	--	--	--	--	--	1	--	--	--	--
2013	Gilmone [35]	1	1	--	--	--	--	--	--	--	--		--	--	--
2013	Kahveci [36]	1	--	--	--	--	--	--	--	--	--	1	--	--	--
2015	Cristaudo [37]	13	10	2	--	--	--	--	--	--	--	1	--	--	--
2015	Our article	10	5	--	--	--	--	--	--	--	--	4	--	--	1
			523	202	58	38	1	2	1	2	1	239	55	1	2

*AT* antibiotic therapy, *AP* appendicectomy, *DV* diverticulectomy, *AP + DV* appendicectomy + diverticulectomy, *AP + EDV* appendicectomy + excision of diverticulum, *SDV* suture of diverticulum, *SC* suture of cecum, *CR* cecum resection, *AP + ACP* appendicentomy + amputation of the cecal pole, *LRH* limited right hemicolectomy, *RH + ITA* Right hemicolectomy and ileo-transverse anastomosis, *ICR + AN* ileocecal resection and anastomosis

In 20 of the studies, the lesions were described intraoperatively. They were perforation of the diverticulum in 42 patients, inflamed cecal wall and perforation in two patients, diverticulum alone in eight patients, cecal mass in 18 patients, right colonic diverticulitis in one patient, and cecal diverticulitis in eight patients (Table 4).

**Table 4:** Intraoperative appearance of the reported cases of solitary cecum diverticulum

Year	Authors	Pt	Perfotated CD	Inflamed cecal wall + perforation	Diverticulum	Cecal mass	Right colonic diverticulitis	inflamed cecal diverticulum
2001	Law [9]	37	NR	NR	NR	NR	NR	NR
2003	Fang [10]	97	NR	NR	NR	NR	NR	NR
2004	Papaziogas [11]	8	8	--	--	--	--	--
2006	Connolly [6]	3	2	--	--	--	--	1
2006	Yang [12]	113	NR	NR	NR	NR	NR	NR
2006	Basili [13]	3	2	--	--	--	--	1
2006	Ruiz-Tovar [14]	5	4	--	1	--	--	--
2007	Griffiths [7]	1	1	--	--	--	--	--
2007	Kurer [15]	1	--	--		1	--	--
2007	Hildebrand [16]	16	12	--	4	--	--	--
2007	Leung [17]	74	NR	NR	NR	NR	NR	NR
2007	Lee [18]	90	NR	NR	NR	NR	NR	NR
2008	Karatepe [19]	4	--	--	2	2	--	--
2009	Kachroo [20]	1	1	--	--	--	--	--
2009	Malek [2]	1	--	1	--	--	--	--
2009	Telem [21]	1		--	--	--	1	
2009	Cole [22]	1	1	--	--	--	--	--
2009	Kumar [23]	2	1	--	--	1	--	--
2009	Butt [24]	1	no	No	no	no	no	no
2010	Matsushima [25]	110	NR	NR	NR	NR	NR	NR
2010	Kim [26]	158	NR	NR	NR	NR	NR	NR
2011	Paramythiotis [27]	1	--	--	1	--	--	--
2011	Radhi [28]	15	4	--	--	7	--	4
2012	Uwechue [29]	1	1	--	--	--	--	
2012	Kwon [30]	59	NR	NR	NR	NR	NR	NR
2012	Issa [31]	15	NR	NR	NR	NR	NR	NR
2011	Tan [32]	68	NR	NR	NR	NR	NR	NR
2013	Tan [33]	226	NR	NR	NR	NR	NR	NR
2013	Kroening [34]	1	--	1	--	--	--	--
2013	Gilmone [35]	1	NR	NR	NR	NR	NR	NR
2013	Kahveci [36]	1	1	--	--	--	--	--
2015	Cristaudo [37]	13	1	--	--	--	--	2
2015	Our article	10	3	--	--	7	--	--
	TOTAL	1138	42	2	8	18	1	8

*Pt* number of patients, *CD* cecal diverticulum, *NR* not reported

Complications were reported for 73 patients (6.4 %), and 56 patients (4.9 %) were reported to have had recurrence of symptoms. In the group of patients with recurrence, 48 patients (85.8 %) were treated with conservative therapy, six patients (10.7 %) underwent AP, and two patients (3.6 %) underwent DV (Table 5).

**Table 5:** Complications and recurrence of solitary cecum diverticulum

Year	Authors	Pt	Complications	Recurrence
2001	Law [9]	37	6	NR
2003	Fang [10]	97	8	NR
2004	Papaziogas [11]	8	NR	NR
2006	Connolly [6]	3	NR	NR
2006	Yang [12]	113	NR	11
2006	Basili [13]	3	No	NR
2006	Ruiz-Tovar [14]	5	NR	NR
2007	Griffiths [7]	1	NR	NR
2007	Kurer [15]	1	NR	NR
2007	Hildebrand [16]	16	No	NR
2007	Leung [17]	74	NR	NR
2007	Lee [18]	90	2	9
2008	Karatepe [19]	4	NR	NR
2009	Kachroo [20]	1	NR	NR
2009	Malek [2]	1	No	NR
2009	Telem [21]	1	NR	NR
2009	Cole [22]	1	NR	NR
2009	Kumar [23]	2	NR	NR
2009	Butt [24]	1	No	NR
2010	Matsushima [25]	110	NR	8
2010	Kim [26]	158	NR	17
2011	Paramythiotis [27]	1	No	NR
2011	Radhi [28]	15	NR	1
2012	Uwechue [29]	1	1	NR
2012	Kwon [30]	59	14	NR
2012	Issa [31]	15	NR	1
2011	Tan [32]	68	26	NR
2013	Tan [33]	226	14	9
2013	Kroening [34]	1	No	NR
2013	Gilmone [35]	1	NR	NR
2013	Kahveci [36]	1	NR	NR
2015	Cristaudo [37]	13	NR	NR
2015	Our article	10	2	NR
	TOTAL	1138	73	56

*Pt* number of patients, *NR* not reported

Histopathologic examination was reported in only 13 studies. The mean length of hospital stay was 4.7 days, and only five studies reported follow-up (Table 6).

**Table 6:** Histopathologic examination and length of hospital stay related to solitary cecum diverticulum

Year	Authors	Pt	HPE	Hospital stay	Follow-up
2001	Law [9]	37	NR	NR	NR
2003	Fang [10]	97	NR	NR	NR
2004	Papaziogas [11]	8	CD	NR	NR
2006	Connolly [6]	3	PD (2 cases)	4,3	NR
2006	Yang [12]	113	NR	NR	NR
2006	Basili [13]	3	1 ID + 2 PD	NR	NR
2006	Ruiz-Tovar [14]	5	NR	NR	NR
2007	Griffiths [7]	1	NR	7	NR
2007	Kurer [15]	1	PD	NR	NR
2007	Hildebrand [16]	16	NR	11,5	NR
2007	Leung [17]	74	NR	5,5	NR
2007	Lee [18]	90	NR	NR	NR
2008	Karatepe [19]	4	NR	NR	NR
2009	Kachroo [20]	1	necrotic SCD	NR	NR
2009	Malek [2]	1	PD	6	NR
2009	Telem [21]	1	NR	4	LRH
2009	Cole [22]	1	PD	7	coloscopy
2009	Kumar [23]	2	PD (1 case) ID (1 case)	4,5	NR
2009	Butt [24]	1	NR	2	NR
2010	Matsushima [25]	110	NR	8	NR
2010	Kim [26]	158	NR	7,4	NR
2011	Paramythiotis [27]	1	SCD	6	coloscopy
2011	Radhi [28]	15	NR	NR	NR
2012	Uwechue [29]	1	PD	NR	port site hernia
2012	Kwon [30]	59	NR	NR	NR
2012	Issa [31]	15	NR	5	NR
2011	Tan [32]	68	NR	NR	NR
2013	Tan [33]	226	NR	NR	NR
2013	Kroening [34]	1	UIM	NR	NR
2013	Gilmone [35]	1	NR	NR	NR
2013	Kahveci [36]	1	CD	NR	NR
2015	Cristaudo [37]	13	NR	3,8	NR
2015	Our article	10	SCD	4,7	NR

Follow-up was not reported in the majority of studies reviewed

*Pt* number of patients, *HPE* histopathologic examination results, *NR*: not reported, *CD* cecal diverticulum, PD:, *LRH* limited right hemicolectomy, *SCD* solitary cecum diverticulum, *UIM*

## Discussion

Diverticulosis is a predominantly Western disease, with a prevalence of 8.5 %[19]. About 50 % of people older than 50 years are affected, and 85 % of these cases occur in the descending and sigmoid colon. Right sided diverticulosis is seen more commonly in the Oriental population, with an incidence as high as 71 %[7]. Cecal diverticulae form 3.6 % of all colonic diverticulae, and 13 % of these develop inflammation at some time [6, 22, 23]. Males are more commonly affected (male:female ratio of 3:2) [6, 22, 23]. The median age at occurrence is 44 years [22, 23]. In our personal experience, the incidence of SCD in relation to appendicitis is as reported in the literature. However, the mean age and male:female ratio found in our study were different to the data reported in the literature. This is probably because Qatar has a high population of expatriate young males working on building the infrastructure.

Cecal diverticulae are classified as congenital or acquired. The congenital cecal diverticulae are true diverticulae; these include all the layers of the cecal wall and develop at 6 weeks gestation from a transient out-pouching of the cecum [6]. The false or acquired diverticulae are similar to sigmoid diverticulae, and contain no muscular layer [6]. Cecal diverticulae can also be classified as solitary or multiple, and can be found in the appendix, cecum, and ascending colon [3]. The solitary cecal diverticulae are usually congenital and true as in our experience, while multiple cecal diverticulae are acquired and false [21].

In about 80 % of cases the cecal diverticulae are positioned 2.5 cm from the ileocecal junction, and about 50 % are on the anterior cecal wall and may cause peritonitis [38]. When the cecal diverticulae are posterior, this may cause inflammatory masses that simulate carcinoma [22]. In our study, none of the masses were due to posterior disease of the cecum wall. This is probably due to the fact that many of these patients arrive in the hospital only in the presence of severe pain that cannot regress with oral treatment, following repeated attacks that may also be due to microperforation causing localized peritonitis that regresses with oral treatment. So in this way repeated attacks can cause a cecal mass to form even if the diverticulum is positioned on the anterior wall of the cecum.

Acute appendicitis is the clinical diagnosis in 85 % of the cases of cecal diverticulitis [6]. In the setting of inflammation, leukocytosis would be characteristic. Clinically, patients with SCD present with a long history of right lower quadrant abdominal pain, with the absence of systemic toxic signs and of nausea/vomiting [39]. Unlike in appendicitis, the pain remains in the right lower quadrant instead of migrating from the epigastrium [40].

Only 10 of 5000 (0.2 %) radiological examinations would diagnose cecal diverticulitis in Oriental people [41]; this improves to 9 % if the patient has had a previous AP [22], and improves further to 65 % intraoperatively [7]. The radiological diagnosis with an abdominal radiograph revealed a fecalith in 50 % of cases, and a barium enema may show the diverticulum as obliteration of its lumen because of surrounding inflammation and edema [38]. Abdominal ultrasound demonstrated a hypoechoic area on a portion of a thickened cecal wall [14]; this radiological procedure has a sensitivity of 91.3 %, specificity of 99.8 %, and an accuracy of 99.5 % for the diagnosis of cecal diverticulitis [42]. CT scans are being increasingly used; this radiological examination showed thickened cecal wall with an extraluminal mass associated with haziness and linear stranding of the pericecal fat [38]. Magnetic resonance imaging can be used in case of equivocal ultrasound features or in case of young or pregnant patients who need to avoid ionizing radiation [43].

Despite advances in these radiological examinations, a correct preoperative clinical diagnosis occurs in only 4–16 % of cases [21]. Between 65 and 85 % of macroscopic diagnosis of SCD is laparoscopic, especially in young females with atypical symptomology [6]. In our case the majority of patients at our hospital are males, which can help with limiting the differential diagnoses. However, in our hospital we usually perform between 7 and 15 surgical procedures per night, and the majority of these are for acute appendicitis; in the absence of specific indications, the diagnosis is based on clinical examination and laboratory testing. In our study, the diagnosis of SCD was made via abdominal CT scan in one patient treated previously with AP, and in nine patients the diagnosis was intraoperative by laparoscopy. As the majority of cases of SCD are treated conservatively, we have chosen to perform the APs to avoid misdiagnosis in case of future inflammation of SCD. This can be useful especially in these patients coming from developing countries where frequently there are not sufficient tools to achieve diagnostic images; the anamnestic record of AP and the diagnosis of SCD can help in choosing the appropriate treatment and reserve surgical treatment for patients with complicated or evolving disease.

There are four grades of diverticulitis according to the management guidelines (ACS recommendations). Grade I: inflamed diverticulum; the treatment is conservative if the diagnosis is made preoperatively, the treatment is AP ± DV if the diagnosis is made intraoperatively. Grade II: inflamed mass. Grade III: localized abscess/fistula. For these two grades the treatment is conservative if the diagnosis is preoperative; if the diagnosis is intraoperative, the treatment is limited ileocecostomy or right hemicolectomy. Grade IV: perforation/ruptured abscess with generalized peritonitis; whether the diagnosis for this grade is pre- or intra-operative, the treatment is limited ileocecostomy or right hemicolectomy [44]. Table 7 lists all treatment possibilities and associated advantages and disadvantages depending on the disease status.

**Table 7:**
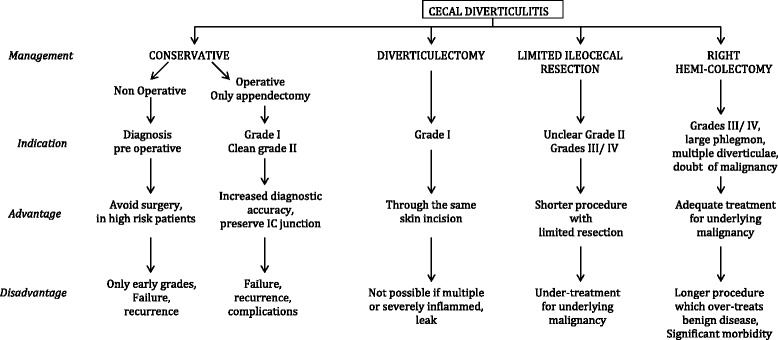
Treatments and associated advantages and disadvantages for different grades of solitary cecum diverticulum

The most commonly reported complications of SCD are inflammation (13 %), bleeding (15 %), hemorrhage, torsion and perforation (12 %) [6].

When diagnosed preoperatively, non-perforated cecal diverticulitis can be managed conservatively with intravenous antibiotics and supportive care with a caution that complicated recurrences are common (up to 20 %) [21]. This therapy can also be used in cases of uncomplicated cecal diverticulitis diagnosed by laparoscopy [22]. A skilled surgeon can conduct a simple DV or invagination of the diverticulum with AP by laparoscopy [6]. In our case, the inflamed wall of the cecum was not treated because residents of fellows or specialists usually perform the procedures during the night and they do whatever is safest for the patients.

Ileocecal resection or right hemicolectomy should be considered in patients with marked inflammation, perforation, or torsion [23]. In cases where the SCD is located on the posterior wall and tumor of the cecum is suspected, right hemicolectomy is mandatory [45]. In a review of 49 patients, 40 % of patients treated with DV or antibiotics alone underwent subsequent hemicolectomy for persistence of the inflammatory process [46]. In another review of 85 patients, less than 40 % were successfully treated with conservative therapy; in the group treated with AP, 29.2 % had a recurrence and 12.5 % were treated subsequently with right hemicolectomy [10]. After surgical treatment for cecal diverticulitis, a mortality of 0 % was reported after ileocecal resection and of 1.8 % after right hemicolectomy [10].

## Conclusion

In conclusion, antibiotic therapy remains the most commonly used treatment for SCD in the literature. In case of operative but conservative treatment, AP is justified to avoid misdiagnosis in case of future episodes of diverticular inflammation.
